# Case Report: Angioimmunoblastic T-cell lymphoma with coexisting plasma cell tumors: three cases and review of the literature

**DOI:** 10.3389/fonc.2025.1705496

**Published:** 2025-11-21

**Authors:** Yulin Yang, Xingshuo Bao, Wei Huang

**Affiliations:** Department of Hematology, Tongji Hospital, Tongji Medical College, Huazhong University of Science and Technology, Wuhan, China

**Keywords:** angioimmunoblastic T cell lymphoma, plasma cell tumors, EBV, case report, lenalidomide

## Abstract

Angioimmunoblastic T cell lymphoma (AITL) is classified as a nodal T-cell lymphoma from T follicular helper(Tfh) cells. Dysfunctional Tfh cells can lead to abnormal B cell regulation and plasma cell differentiation in AITL. However, the coexistence of AITL with plasma cell tumors is exceedingly rare. Here, we report three patients diagnosed with AITL accompanied by monoclonal gammopathy of undetermined significance (MGUS), smoldering multiple myeloma (SMM), and multiple myeloma (MM). The clinicopathologic findings, together with a review of previously reported cases, provide insight into the possible biological relationship between AITL and plasma-cell neoplasms. Epstein–Barr virus (EBV) infection was detected in all cases, suggesting a potential—though unproven—association between EBV-driven immune dysregulation and plasma-cell proliferation. Case analysis showed that some monoclonal plasma cells originated from the interior of AITL, while others originated from the bone marrow. This indicated that the evolution of plasma cell tumors may arise from either the lymphoma microenvironment or the systemic immune status. In cases of coexistence of AITL and plasma cell tumors, the treatment containing anti-MM drugs such as lenalidomide was usually recommended and might bring therapeutic benefits. Careful identification of monoclonal plasma-cell populations is therefore important for accurate diagnosis and individualized management of AITL.

## Introduction

Angioimmunoblastic T cell lymphoma (AITL) is classified as a nodal T-cell lymphoma with follicular helper T-cell (Tfh) phenotype, which is a unique subtype of peripheral T cell lymphoma(PTCL). AITL accounted for 15-20% of PTCL (1). This disease mainly affects the middle-aged and older population, with a median diagnosis age of about 65 years old. More than 75% of AITL patients were diagnosed in advanced stages and had a poor prognosis (2). AITL patients often tested positive for autoimmunity and presented a general increase in immunoglobulins (3).

The cell of origin for AITL lymphoma was the Tfh, which coincided with the autoimmune features and B-cell proliferation in AITL tissue. Recent studies have emphasized the close relationship between the AITL tumor microenvironment and the abnormal proliferation of Tfh cells. The characteristics of the AITL tumor microenvironment included the proliferation of high endothelial venules (HEVs), complex network structures of follicular dendritic cells (FDCs), and infiltration of various reactive cell types, including fibroblasts, germinal center B cells, and dendritic cells. The intense interaction between Tfh cells and germinal center B cells might lead to dysregulation of B cells, resulting in immune disorders such as autoimmune hemolytic anemia, plasma cell proliferation, or polyclonal hypergammaglobulinemia (4). The increased expression levels of inflammatory/anti-inflammatory cytokines and the characteristic vascular proliferation reflected the AITL enriching inflammation signature (5).

Chronic inflammation plays a crucial role in the development of tumors. In the AITL tumor microenvironment, the dysfunction of Tfh cells and the imbalance of cytokines led to the proliferation of B cells and plasma cells (4). Infiltrated B cells and plasma cells might develop into monoclonal B cells and plasma cells under DNA damage and successfully grow into tumors through immune escape mechanisms. The Epstein-Barr virus(EBV)-positive B cells were detected in 66–86% of patients with AITL, and a strong correlation between EBV-infected B cells and AITL pathogenesis was established (6). Previous reports showed 19 cases of B-cell non-Hodgkin lymphoma among 161 cases of AITL diagnosed between 1996 and 2005 (7). Cases of AITL with coexisting plasma cell tumors were rarely reported. The behavior of plasma cells, which ranged from reactive plasmacytosis to striking clonal proliferation, has not yet been deciphered in AITL (8).

We report 3 cases of AITL with coexisting plasma cell tumors, including Monoclonal gammopathy of undetermined significance (MGUS), smoldering multiple myeloma (SMM), and multiple myeloma (MM). These cases reflected the evolution of plasma cell clones and the possible association with EBV virus infection during the development of AITL. We completed a narrow literature review to gain a deeper understanding of the behavior of plasma cells in AITL.

## Case presentation

### Case 1

A 54-year-old male patient was admitted to the hematology department of our hospital (Tongji Hospital, Tongji Medical College, Huazhong University of Science and Technology)because of relapsing fever and bilateral lower limb skin rash for three months in October 2021. The patient denied bone pain, night sweats, and significant weight loss. His past medical history included cervical lymphadenitis, which was cured after anti-infection treatment in April 2021. Physical examination revealed cervical, axillary, and inguinal lymphadenopathy, splenomegaly, and a bilateral lower extremity rash. The laboratory data showed anemia [91g/L (normal: 115–150g/L)], normal renal function with proteinuria, high serum lactic dehydrogenase [278 U/L, (normal: 120–250 U/L)], low serum albumin [28g/L, (normal: 40–55g/L)], abnormal erythrocyte sedimentation rate [25mm/H (normal: 0–20mm/H)]. The overall autoimmune workup revealed a high titer of antinuclear antibodies and low complement C3. Traditional detection of pathogenic microorganisms in the blood was negative, except for Epstein-Barr virus (EBV). Serological tests for EBV showed positive for viral capsid antigen (VCA) immunoglobulin G (IgG) and viral nuclear antigen (VNA) IgG antibodies, while negative for VCA-IgM. PCR detection showed an increase in the copy number of EBV in plasma and peripheral blood mononuclear cells (PBMC), and EBV sorting PCR showed EBV mainly infected T and B cells (**Table 1**). Monoclonal gamma globulin identification showed IgG-λ M protein. The quantitative results from serum protein electrophoresis showed that the absolute value of serum M protein was 2.2g/L (normal: 0g/L). The quantitative results from serum immunofixation electrophoresis showed normal levels of IgA [3.91g/L, (normal: 0.82–4.53g/L)], IgG [15.3g/L, (normal: 7.51–15.6g/L)], IgM [1.66g/L, (normal: 0.46–3.04g/L)] and abnormal levels of serum free light chain κ [138.6mg/L, (normal: 3.30–19.40mg/L)], serum free light chain λ[155.2mg/L, (normal: 5.71–26.30mg/L)], κ/λ[0.098, (normal: 0.26–1.65)]. The level of beta-2 microglobulin [10.83mg/L (normal: 0.8–2.2mg/L)] increased significantly. Then, bone marrow tests were carried out. The flow cytometric analysis of bone marrow revealed 0.88% mature T cells with abnormal phenotypes (CD45+、CD2+、CD5+、CD45RO+、CD200+、PD1hi+、CD4dim+、CD3-、CD7-、CD56-、CD8-、CD45RA-、CD57-、CD30-、TCRab- 、TCRrd- 、CD103-、CD25-、TRBC1-、CD10-、CD26-、Ki67 LI (about 25.6%). The flow cytometric analysis of bone marrow revealed a normal plasma cell phenotype. The bone marrow PCR assay, as described in the European BIOMED−2 collaborative study (9), indicated the presence of clonal rearrangements of TCR instead of Ig.

**Table 1:** Laboratory test data during diagnosis of three cases.

Parameter	Case 1	Case 2	Case 3
White blood cells (3.5-9.5*10^9/L)	5.56	5.45	5.06
Hemoglobin (130-175g/L)	91	102	90
Platelets (125-350*10^9/L)	116	241	158
Globulin (20-35g/L)	33	63.8	43.7
Albumin (40-55g/L)	28	30.6	37.1
LDH (135-225U/L)	278	160	194
Serum creatinine (45--84umol/L)	78	74	55
hypercalcemia	no	no	no
bone destruction	no	no	no
qualitative urine protein (negative)	positive	negative	negative
copy number of EBV in plasma(<5*10^2/mL)	1.4*10^3	9.36*10^2	negative
copy number of EBV in PBMC(<5*10^2/mL)	2.98*10^5	5.97*10^4	5.05*10^3
EBV sorting PCR			
T (undetected/2*10^5)	4.710*10^6	NA	undetected
B (undetected/2*10^5)	3.973*10^6	NA	2.891*10^5
NK (undetected/2*10^5)	6.970*10^3	NA	9.121*10^2
PBMC (undetected/2*10^5)	9.147*10^4	NA	3.151*10^4
IL-1β(<5pg/mL)	10	NA	NA
sIL-2R(223-710U/mL)	>7500	NA	NA
IL-6(<7pg/mL)	138.8	NA	NA
IL-8(<62 pg/mL)	1581	NA	NA
IL-10(<9.1 pg/mL)	59.4	NA	NA
TNFα(<8.1 pg/mL)	90.5	NA	NA
M protein (type,Normal value:0 g/L)	IgG-λ,2.2	IgG-λ,43.8	IgA-λ,11.8
Bone marrow monoclonal plasma cells	negative	negative	positive
IgH Cloning identification in bone marrow	negative	negative	NA
Plasmacytic differentiation in AITL	negative	positive	negative
Light chain restriction of plasma cell in AITL	negative	positive	negative
IgH Cloning identification in AITL	NA	positive	NA
EBER CISH in AITL	Positive(slightly)	positive(slightly)	positive(slightly)
Diagnosis*	AITL and non-IgM MGUS	AITL and SMM	AITL and MM

PBMC, peripheral blood mononuclear cells; NA, Not detected.

*All three cases were diagnosed simultaneously before anti-tumor treatment. EBV sorting PCR was performed only at diagnosis. Post-treatment B/T-cell infection ratios were not available due to limited archived samples.

**Figure 1:**
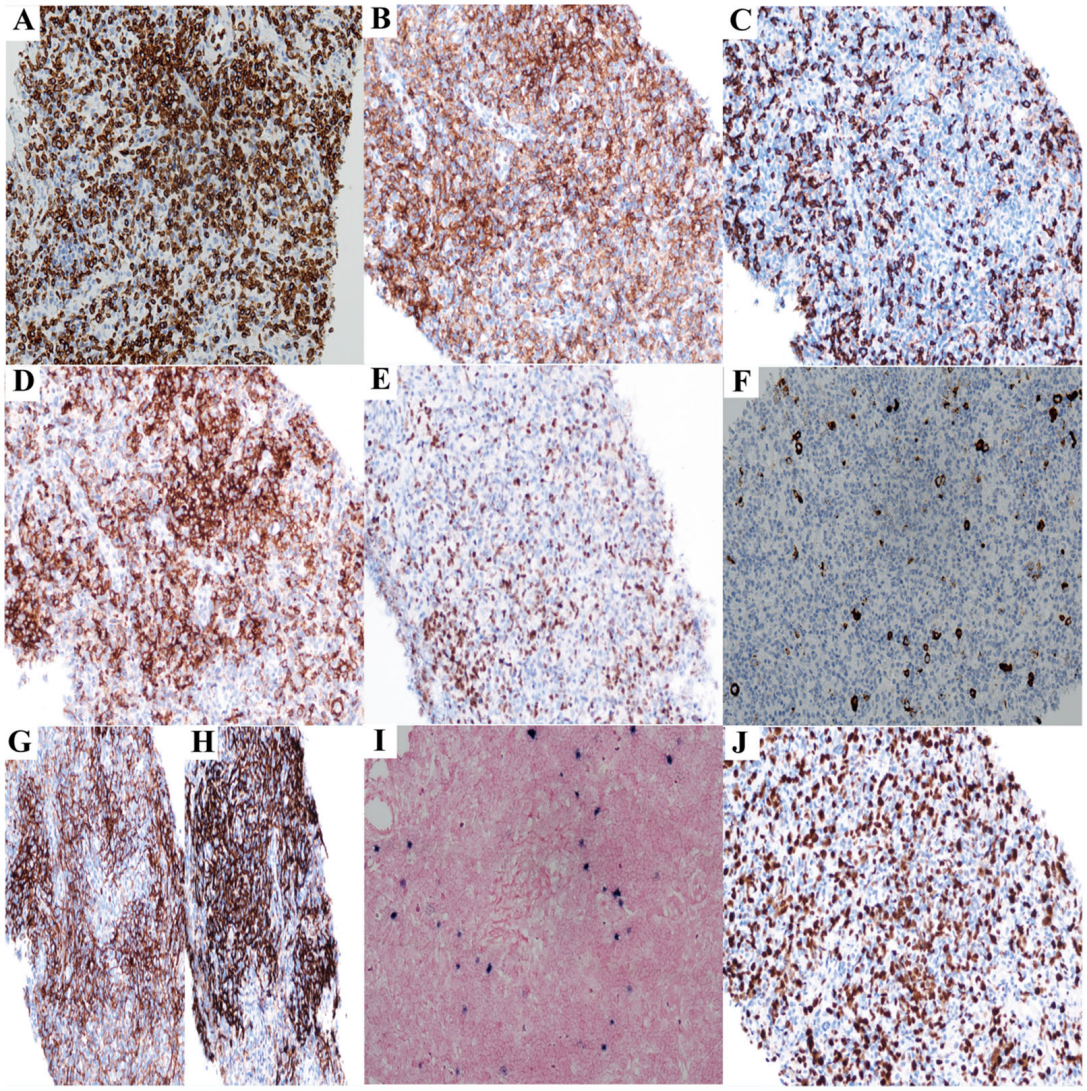
The immunohistochemistry results of lymph node biopsy (Case1) (All images original magnification: ×200). **(A-E)** Tumor cells show extensive positive expression of CD3 **(A)**, CD4 **(B)**, and PD1 **(D)**; scattered cells express CD8 **(C)** and Bcl6 **(E)**. **(F)** CD30-positive cells account for 5% of the total cells. **(G, H)** CD21 **(H)** and CD35 **(G)** reveal irregular proliferation of the FDC network. **(I)** EBER is expressed in a small number of cells. **(J)** The Ki67 proliferation rate is approximately 60%.

After anti-infective treatment, his fever remained. Positron emission tomography-computed tomography (PET-CT) was performed. PET-CT showed multiple enlarged hypermetabolic lymph nodes in bilateral neck (SUVmax, 3.6), bilateral supraclavicular region (SUVmax, 5.4), bilateral axillary (SUVmax, 5.0), mediastinum (SUVmax, 4.8), retroperitoneum (SUVmax, 4.4), bilateral iliac vessels (SUVmax, 3.7) and splenomegaly (SUVmax, 3.9). We performed a needle biopsy of the enlarged lymph node on the right supraclavicular region. The immunohistochemistry results showed CD3+、PD1+、BCL-6+、CD4+、CD8+、irregular proliferation of follicular dendritic cell (FDC) network defined by CD21/35 staining、CD30 (VENTANA)(+, positive cells/total cell count of approximately 5%, positive control+)、Ki67 LI:about 60%、EBER (CISH) (slightly+) (**Figure 1**). EBER(CISH) is intended to detect EBV RNA by chromogenic *in situ* hybridization. The tumor samples’ PCR assay (9) indicated the presence of clonal rearrangements of TCR. The pathological diagnosis was AITL. The patient was eventually diagnosed with AITL and Non-IgM MGUS based on diagnostic criteria (10) on 7 November 2021, and the patient received CHOP therapy. After two CHOP therapy courses, PCR detection showed that the copy number of EBV in plasma turned negative. Subsequently, the patient completed six cycles of CHOP therapy. The patient was followed up regularly every 1–3 months via outpatient clinic visits. Laboratory tests including serum M protein levels, EBV viral load, and imaging studies were performed at each visit. After six cycles of CHOP chemotherapy, the patient’s M protein level dropped to 0 g/L, and EBV DNA in plasma became undetectable. However, six months post-therapy, PET-CT revealed lymphadenopathy suggestive of relapse. The patient received GEMOX+selinexor regimen with disease progression, followed by PD-1 inhibitor combined with SMILE chemotherapy. Due to poor tolerance, the patient transitioned to traditional Chinese medicine and monthly PD-1 inhibitor therapy. As of December 2024, the patient remains under stable observation with signs of partial lymphoma shrinkage.

### Case 2

A 60-year-old female patient was admitted to the otolaryngology department of our hospital(Tongji Hospital, Tongji Medical College, Huazhong University of Science and Technology) because of a right cervical lymph node enlargement for two months in May 2023. The patient denied bone pain, fever, night sweats, and significant weight loss. Her past medical history included left upper lobe bronchiectasis, which was ineffective after anti-infection treatment in May 2021. The physical examination showed swollen lymph nodes in the right neck. The blood biochemistry and routine blood test showed normal, except for anemia [102g/L (normal: 115–150g/L)], increased globulin[63g/L(20-35g/L)] and decreased albumin[30.6g/L (40-55g/L)]. The overall autoimmune workup revealed normal results, except for the high titer of antinuclear antibodies. Serological tests for EBV showed positive for VCA-IgG and VNA-IgG antibodies while negative for VCA-IgM. EBV PCR detection showed an increase in the copy number of EBV in plasma and PBMC (**Table 1**). The overall autoimmune workup revealed a high titer of antinuclear antibodies. Monoclonal gamma globulin identification showed IgG-λ M protein. The quantitative results from serum protein electrophoresis showed that the absolute value of serum M protein was 43.8g/L (normal: 0g/L). The quantitative results from serum immunofixation electrophoresis showed normal levels of IgA [1.44g/L, (normal: 0.82–4.53g/L)], IgM [1.61g/L, (normal: 0.46–3.04g/L)], abnormal levels of IgG [44.3g/L, (normal: 7.51–15.6g/L)] and abnormal levels of serum free light chain κ [47.14mg/L, (normal: 3.30–19.40mg/L)], serum free light chain λ[272.97mg/L, (normal: 5.71–26.30mg/L)], κ/λ[0.173, (normal: 0.26–1.65)]. The level of beta-2 microglobulin [6.80mg/L (normal: 0.8–2.2mg/L)] increased significantly. Then, bone marrow tests were carried out. The flow cytometric analysis of bone marrow did not detect any abnormal T or plasma cell clones.

**Figure 2:**
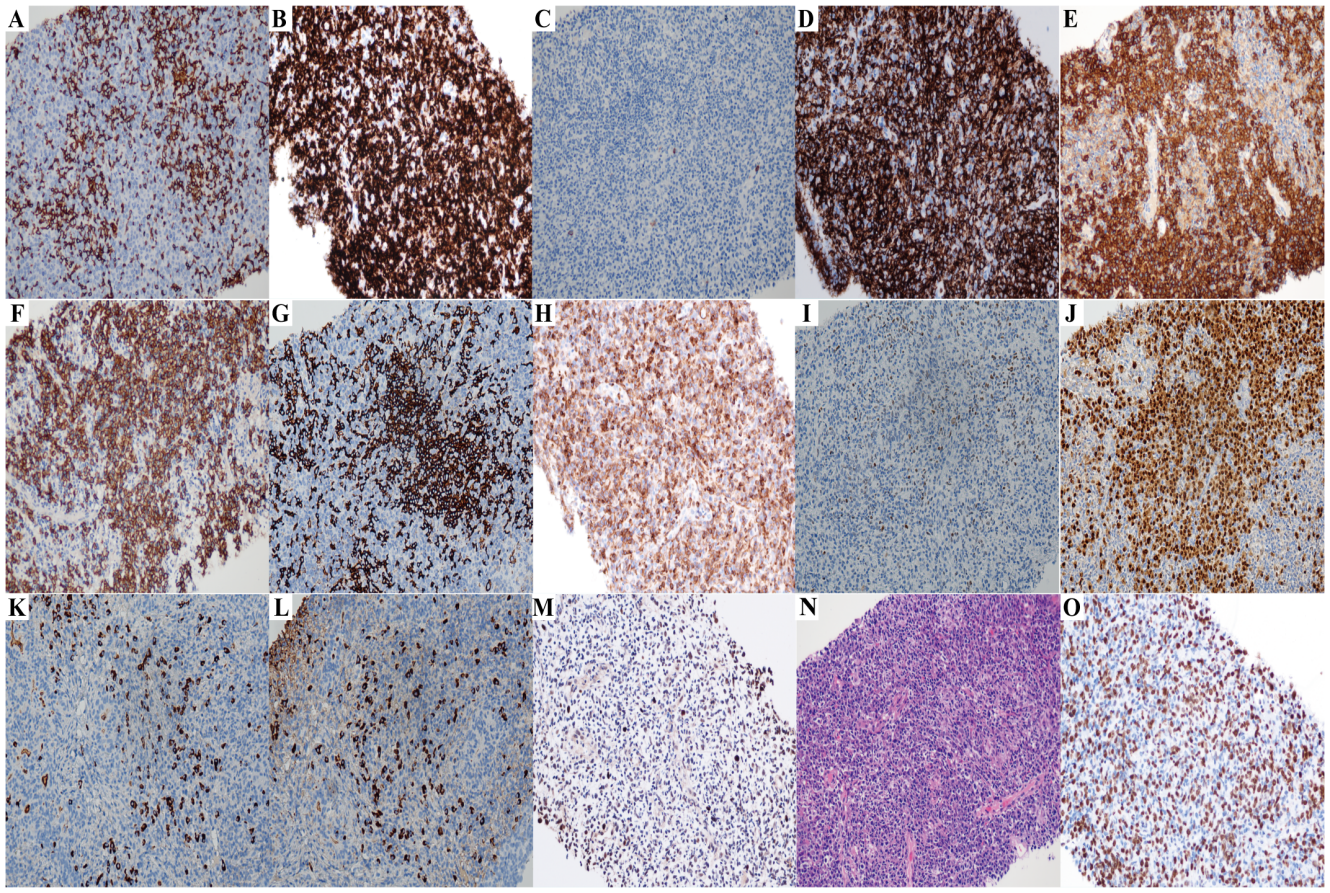
The immunohistochemistry results of lymph node biopsy (Case2) (All images original magnification: ×200). **(A-D,G-I)** Tumor cells commonly express CD3 **(A)**, CD5 **(B)** and CD43 **(D)**, while some also express BCL2 **(H)** and a few BCL6 **(I)**; CD10 **(C)** and CD20 **(G)** are negative. **(E, F, J)** Plasma cell immunostaining: CD38 **(E)**, CD138 **(F)**, and MUM1 **(J)** are positive. **(K, L)** λ light chain **(L)** is significantly more abundant than κ light chain **(K)**, indicating λ light chain restriction. **(M)** EBER is expressed in a small number of cells. **(N)** Hematoxylin and eosin staining reveals histological features of AITL. The lymph node architecture is effaced by a polymorphic infiltrate, with the prominent proliferation of arborizing high endothelial venules (HEVs), and perivascular aggregation of neoplastic clear cells is observed. There is a small amount of plasma cell distribution in it. **(O)** The Ki67 proliferation rate is approximately 50%.

Chest and abdominal CT scans showed enlarged lymph nodes in the bilateral cervical, axillary, and mediastinum (**Supplementary Figures 3A-D**). We performed a needle biopsy of the enlarged lymph node on the right supraclavicular region. The immunohistochemistry results showed tumor cells CD3(+)、CD5(+)、CD43(+)、BCL2(Partial+)、BCL6(scattered weak+)、CD10 (–)、irregular proliferation of follicular dendritic cell (FDC) network defined by CD21/35 staining、CD20(-)、CD20(positive control+)、CD19(-)、CD22(-)、CD79a(-)、PCK(-)、P53 (scattered+, wild-type)、 CyclinD1(-)、IgD(-)、Ki-67 LI:50% in hot spot、plasma cells CD38(+)、CD138(+)、MUM1(+)、λ(+)>>κ(+, monoclonality)、EBER CISH(individual moderate cells+) (**Figure 2**). The tumor samples’ PCR assay (9) indicated the presence of clonal rearrangements of IgH and TCR. The pathological diagnosis was AITL and clonal plasma cell proliferation.

The patient was eventually diagnosed with AITL and SMM based on diagnostic criteria (10) on 20 May 2023, and the patient received lenalidomide+COP therapy. She was lost to follow-up after one cycle of lenalidomide+COP therapy due to personal reasons. This precluded assessment of long-term treatment response, M-protein dynamics, and imaging changes. This represents a significant limitation in evaluating the efficacy of lenalidomide-based regimens in this rare composite lymphoma.

### Case 3

**Figure 3:**
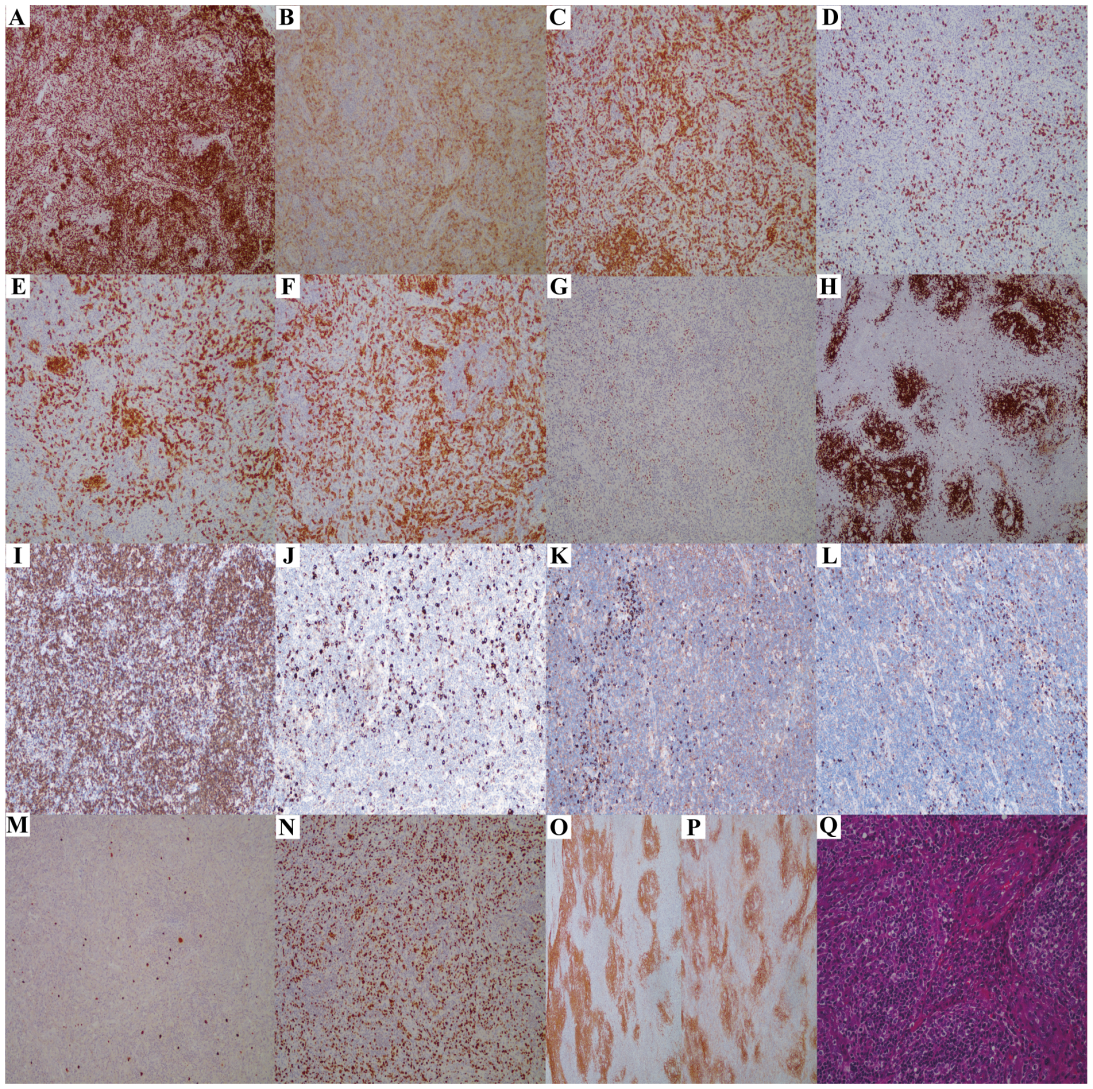
The immunohistochemistry results of lymph node biopsy (Case3). **(A-I)** Tumor cells show widespread expression of CD3 **(A)**, CD5 **(C)**, and Bcl2 **(I)**; scattered positivity for CD4 **(B)**, PD1 **(F)**, and Bcl6 **(G)**; and negativity for CD8 **(D)**, CD10 **(E)**, and CD20 **(H)** (×100 magnification). **(J)** CD30-positive cells represent 5% of the total cells(×100 magnification). **(K, L)** Plasma cells show slight positivity for lambda **(K)** and kappa **(L)**, with no light chain restriction(×100 magnification). **(M)** EBER positivity is noted in individual cells(×100 magnification). **(N)** Ki67 indicates a high rate of cell proliferation, approximately 80%(×100 magnification). **(O, P)** CD21 **(O)** and CD35 **(P)** reveal irregular proliferation of the FDC network (×100 magnification). **(Q)** Hematoxylin and eosin staining reveals the histological characteristics of AITL, with a significant number of high endothelial venules (HEVs) proliferating (×200 magnification).

A 75-year-old female patient was admitted to the otolaryngology department of our hospital(Tongji Hospital, Tongji Medical College, Huazhong University of Science and Technology) because of a cervicalmass of one-week duration in September 2021. The patient denied bone pain, fever, night sweats, and significant weight loss. Her past medical history included an appendectomy due to appendicitis. The physical examination showed the cervical swollen lymph nodes and the right nasopharyngeal wall mass. The blood biochemistry and routine blood test showed normal. The patient underwent a nasopharyngeal mass biopsy under nasal endoscopy. The pathological diagnosis was atypical lymphocytic proliferation. One month later, the patient underwent cervical mass resection surgery again in the otolaryngology department due to enlargement of the cervical mass. The immunohistochemistry results showed tumor cells CD3(+)、CD5(+)、CD43(+)、LCA(+)、PD1(scattered weak+)、BCL6(scattered weak+)、CD4(scattered +)、C-MYC(+)、BCL2(+)、CD8 (–), plasma cells MUM(+)、λ(a little +)、κ(a little +), CD30 (approximately 5% moderate intensity+, positive control+)、GATA3 (slight+)、 P53 (scattered+, indicating wild-type)、 CD21 and CD23 (residual FDC network+)、CD35 (irregular proliferation of small focal FDC network+)、 CD20 (-, positive control+)、CD19(-)、PAX5(-)、CD10(-)、CyclinD1(-)、 PCK (-) Ki -67(LI about80%), EBER CISH (individual+, positive control+) (**Figure 3**). The tumor samples’ PCR assay [9] indicated the presence of clonal rearrangements of TCR. The patient was eventually diagnosed with AITL and admitted to the hematology department. The blood biochemistry and routine blood test showed normal, except for anemia [90g/L (normal: 115–150g/L)]. EBV PCR detection showed an increase in the copy number of EBV in PBMC, and EBV sorting PCR showed that EBV mainly infected B cells (**Table 1**). Monoclonal gamma globulin identification showed IgA-λ M protein. The quantitative results from serum protein electrophoresis showed that the absolute value of serum M protein was 11.8g/L (normal: 0g/L). The quantitative results from serum immunofixation electrophoresis showed elevated levels of IgA [14.9g/L, (normal: 0.82–4.53g/L)], decreased levels of IgM [0.43g/L, (normal: 0.46–3.04g/L)] and IgG [44.3g/L, (normal: 7.51–15.6g/L)], normal levels of serum free light chain κ [10.2 mg/L, (normal: 3.30–19.40mg/L)], serum free light chain λ[24.1mg/L, (normal: 5.71–26.30mg/L)], κ/λ[0.43, (normal: 0.26–1.65)]. Then, bone marrow tests were carried out. Bone marrow aspiration revealed 21% immature plasma cells. The flow cytometric analysis of bone marrow revealed abnormal plasma cells with phenotypic abnormalities (CD38dim+, CD138+, clambda +, CD45-, CD20-, CD19-, CD56-, ckappa-). The flow cytometric analysis of bone marrow also detected 0.02% abnormal T cell clones with phenotypic abnormalities (CD45+, CD4+, CD5bri+, CD45RO+, PD1bri, CD26bri, Ki69 9.4%, CD3-, CD7-, CD8-, CD45RA-, CD200-, CD57-, CD30-, TCRab-, TCRrd-, CD103-, CD25-, TRBC1-, CD10-) (**Supplementary File 2**).

The results of bone marrow fluorescence *in situ* hybridization (FISH) showed that the patient had 1q21 amplification, IGH gene amplification, CCND gene amplification, and +17 chromosome (**Supplementary Figure 2**). The patient was eventually diagnosed with AITL and MM (10) on 19 November 2021. The patient received 5 courses of therapy (lenalidomide+CHOP). PET evaluation in April 2022 showed regression of lymphoma tumor. The quantitative results from serum protein electrophoresis showed the absolute value of serum M protein was 7.4g/L (normal: 0g/L). From May 2022, the patient received five courses of therapy (VRD). Bone marrow cytology and flow cytometry did not detect any abnormal T or plasma cell clones. The quantitative results from serum protein electrophoresis showed the absolute value of serum M protein was 0 g/L (normal: 0g/L). The results from serum immunofixation electrophoresis were normal. Patients took oral lenalidomide for maintenance treatment. As of December 2024, the patient is still alive.

## Discussion

AITL is the nodal T-cell lymphoma originating from Tfh. The Tfh are a subset of CD4+ T cells specialized to regulate antibody responses. Tfh cells help B cells form germinal centers (GC) to differentiate into memory cells and plasma cells (antibody-secreting cells) as inflammatory responses (11). AITL tumor microenvironment might include abundant B cells and plasma cells (12), but plasma cells were usually polyclonal rather than monoclonal in a few cases (13). So, cases of AITL with coexisting plasma cell tumors were rarely reported.

MM is a common hematologic malignancy involving malignant plasma cells that can occur not only in the bone marrow but also in extramedullary tissue. MM is universally preceded by precursor stages clinically termed as MGUS and SMM (10). We reported three cases of AITL with coexisting plasma cell tumors. These cases were AITL combined with MGUS, AITL combined with SMM, and AITL combined with MM. All three cases were diagnosed almost simultaneously, and none of them used chemotherapeutic drugs before diagnosis. For case 1 of AITL with coexisting MGUS, there were no monoclonal plasma cells in the bone marrow and AITL samples, except for the M protein. MGUS did not receive more attention because of slow progression to MM and undamaged organs. The patient received CHOP regimens, which did not include common drugs for treating MM, such as bortezomib and lenalidomide. After treatment, M protein could be cleared. For case 2 of AITL with coexisting SMM, there were monoclonal plasma cells in AITL samples and M protein, but there were no monoclonal plasma cells in the bone marrow. The level of M protein far exceeded the diagnostic criteria for SMM (10). The patient was treated with a regimen containing lenalidomide. For case 3 of AITL with coexisting MM, there were 21% monoclonal plasma cells in the bone marrow and M protein, but there were no monoclonal plasma cells in AITL samples. The bone marrow fluorescence *in situ* hybridization (FISH) results showed that the patient had 1q21 amplification, IGH gene amplification, CCND gene amplification, and +17 chromosome (**Supplementary Figure 2**). The patient was also treated with a regimen containing lenalidomide and continued to receive lenalidomide maintenance after treatment.

EBV was associated with AITL in more than 80% of cases and was thought to promote immune escape and survival of infected AITL cells (14). EBV predominantly latently infects activated B cells in AITL tissues and exhibits latent and clonal proliferation (15). EBV latent membrane proteins (LMPs) may play pivotal roles in shaping the tumor microenvironment of AITL. LMP1 functions as a constitutive mimic of CD40 signaling, activating the NF-κB and JAK/STAT pathways, while LMP2A acts as a surrogate B-cell receptor (BCR) that provides chronic survival signals even in the absence of antigenic stimulation (16). Through these mechanisms, LMP2A simultaneously down-regulates MHC class II expression, enabling EBV-infected B cells to evade immune recognition (17). Together, LMP1 and LMP2A drive the secretion of IL-10 and other immunomodulatory cytokines, fostering an immunosuppressive milieu that supports plasma-cell differentiation and survival (18, 19). Persistent expression of these latent genes is closely associated with genomic instability and epigenetic dysregulation. EBV-encoded proteins can induce DNA-damage responses, impair DNA-repair pathways, and alter chromatin remodeling, thereby predisposing infected B cells to malignant transformation (19–21). Within the AITL microenvironment, EBV-positive plasma cells may thus represent a latency-driven proliferative compartment characterized by cumulative genetic and epigenetic insults. Importantly, EBV-encoded proteins further contribute to tumorigenesis by inducing mutations, disrupting cell-cycle regulation, and establishing aberrant methylation patterns that promote plasma-cell survival and expansion (20, 22, 23). The TET2 and DNMT3A gene, as common epigenetic regulators, often underwent mutations in both AITL and clonal hematopoiesis (CH) in the hematopoietic system, and TET2/DNMT3A mutations can also be detected in MM, albeit rarely (24–26). The same TET2 mutation was also detected in bystander B cells of AITL patients as in AITL tumor T cells, suggesting a possible shared clonal origin or cross-lineage evolution (27). Taken together, these findings suggest that EBV-driven genomic instability cooperates with pre-existing TET2/DNMT3A mutations to facilitate the emergence of monoclonal plasma-cell populations within AITL.

In addition to their intrinsic survival advantage, these EBV-positive B cells can present viral antigens to tumor Tfh cells via MHC class II molecules and provide co-stimulatory signals, thereby activating tumor T cells. Simultaneously, EBV-induced cytokine alterations and surface molecular changes inhibit CD8+ cytotoxic T cells, impairing the immune surveillance of EBV-positive B cells, allowing them to evade immune clearance and proliferate abnormally, often in a polyclonal manner (28). Through this reciprocal interaction, EBV-positive B cells and neoplastic T cells form a symbiotic interaction: EBV provides proliferation stimulation for B cells, while B cells provide antigens and co-stimulatory signals to TFH tumor cells, promoting tumor cell survival and proliferation. Over time, this bidirectional signaling may promote progressive immune dysregulation and chronic antigenic stimulation, setting the stage for monoclonal plasma-cell expansion and eventual plasma-cell neoplasm development. Indeed, different EBV subtypes appeared to be associated with post-diagnosis survival (15), suggesting biological heterogeneity in EBV-driven transformation pathways.

In the rare case of AITL combined with plasma cell tumors, EBV may influence the behavior of plasma cell clones indirectly. High levels of pro-inflammatory cytokines (e.g., IL-6, IL-21, etc.) can occur in AITL patients due to EBV-associated immune dysregulation (29). Among them, IL-6 from dendritic cells and macrophages serves as a pivotal factor in promoting plasma cell proliferation within the AITL microenvironment. The IL-6 not only stimulates the proliferation of Tfh tumor cells but also imparts pro-survival and anti-apoptotic signals to adjacent plasma cells, thereby promoting the clonal expansion of plasma cells (29). Mechanistically, IL-6 activates the STAT3 pathway, leading to upregulation of anti-apoptotic proteins such as BCL-xL and BCL-2 in neighboring plasma cells (30, 31). In addition, persistent IL-6/STAT3 signaling can aberrantly reactivate BCL6 expression in IL-6–dependent myeloma cells, fostering uncontrolled proliferation and treatment resistance (32, 33). Consequently, dysregulated IL-6/STAT3/BCL6 signaling—further amplified by EBV-induced inflammation—may represent a biological link between AITL and coexisting plasma-cell neoplasms, providing a rationale for the emergence of plasma-cell clones both within AITL lesions and in the bone marrow compartment.

Clinically, EBV involvement was evident in our cohort: there were not only positive EBER CISH in AITL samples but also positive EBV DNA load in the PBMC. In two cases of EBV sorting PCR test, B cells were the main cells infected by EBV. This suggested that the evolution of plasma cell tumors in the AITL environment may be driven by B cell-mediated mechanisms. However, our ability to assess the dynamic role of EBV infection was limited by the available data. EBV sorting PCR was performed only at initial diagnosis, and post-treatment B/T-cell infection ratios were not available because archived samples were insufficient for repeat analysis. This limitation underscores the need for future studies incorporating serial EBV quantification and cell-type–specific analyses to better clarify the temporal relationship between EBV reactivation and plasma-cell evolution in AITL. Notably, plasma EBV DNA was detectable in Case 1 but absent in Case 3. Case 1 relapsed after six months of CHOP chemotherapy and had multiple relapses, while Case 3 presented with persistent remission of AITL. These contrasting outcomes are consistent with prior studies reporting that elevated EBV DNA load may reflect chronic active infection and portend poorer prognosis in peripheral T-cell lymphomas (34). Moreover, in the study of the relationship between mature B cell neoplasms and the infection, Bosseboeuf A found that EBV was the most frequent initiating factor of plasma cell neoplasms (MGUS, SMM, MM) and EBV was more involved in the development of MGUS and SMM rather than MM (35, 36). Together, these molecular and clinical findings support a model in which EBV infection, through combined genetic, epigenetic, and immunologic mechanisms, contributes to plasma-cell tumor evolution within the AITL microenvironment. A comprehensive literature search was conducted using the PubMed database (from inception to December 2024) with the following search strategy: (“angioimmunoblastic T-cell lymphoma” OR “AITL”) AND (“plasma cell” OR “multiple myeloma” OR “MGUS” OR “plasmacytosis” OR “monoclonal gammopathy”). Reports written in English describing concurrent or sequential AITL and plasma-cell disorders were included. Through searching literature from the PubMed, we retrieved 10 cases of AITL with coexisting plasma cell tumors (37–45) (**Table 2**). All cases tested positive for EBERs, which supported the involvement of EBV in the MM evolution in AITL cases. Only 3 cases were diagnosed with MM combined with AITL (2 cases of MM, 1 case of SMM). The 7 cases were still on the way to MM. There were 4 cases of clonal plasma cell proliferation diagnosed in AITL tissues. The other three cases showed M protein with or without corresponding organ damage (MGUS, MGRS, POEMS). We analyzed these 10 retrieved cases in conjunction with the 3 cases in the text for commonalities and differences, The expression of CD138 was consistent in most cases; high percentage of light chain restriction of lambda chain and IgG type M proteins; EBERs were positive in some cases, indicating that EBV may play a certain role in pathological progression. EBV-positive patients may show more immune escape characteristics, such as increased expression of Bcl6 and PD1, indicating a limitation in the immune system’s ability to recognize and eliminate tumor cells. However, the clinical manifestations of these patients vary widely, such as the presence of B symptoms and concomitant skin rashes. The treatment strategies employed for the patients differed, and accordingly, their therapeutic responses exhibited variation. It might be related to the fact that the type of monoclonal plasma cells (e.g., IgG, IgA, IgM) and the expression of immunophenotypic markers (CD56, PD1, etc.) have an important impact on the clinical prognosis of the patients.

**Table 2:** Cases of plasma cell tumors associated with AITL from literature.

Case no.	Sex /age (y)	Diagnosis	Rash	B-symptom	M protein (quantification)	Site (%)	Igh /ig chain restriction	EBERs	CD56 /CD138 / bcl6 /PD1	Treatment	Follow up(mo)	Reference
1	70/F	CPCP	NA	NA	Neg	LN ,NA	MC/lambda	NA	NA/NA/NA/NA	RCHOP	AWD (39)	(31)
2	56/M	CPCP	Pos	NA	Neg	LN ,NA	MC/lambda	NA	NA/NA/NA/NA	NA	NA	(31)
3	77/F	CPCP	Pos	Pos	Neg	LN,NA	MC /lambda	Pos (T,B)	NA/NA/+/+	NA	DD (8)	(34)
4	61/M	CPCP	NA	NA	Neg	LN ,NA	OC/NA	Pos (B,P)	NA/NA/NA/NA	NA	AWD (24)	(29)
5	79/F	MGUS	Pos	Pos	IgG-κ (1.5g/L)	Neg	NA/kappa	NA	NA/NA/NA/NA	CHOP	DD (12)	(28)
6	70/M	MGRS	Neg	Neg	IgM-λ (1.05g/L))	Neg	NA/lambda	NA	NA/NA/NA/NA	CHOP	NA	(30)
7	53/M	POEMS	Pos	Pos	IgG-λ (23.9g/L)	Neg	NA/lambda	NA	NA/NA/NA/NA	GEMOX +L−asp	NA	(33)
8	87/F	SMM	Neg	Pos	IgG-κ (3.58g/L)	BM, 16.7%	MC/ kappa	Pos (T,B)	+/+/+/+	RCHOP+Lenalidomide	DD (12)	(35)
9	80/M	MM	Neg	Pos	IgA-λ (2.98g/L)	BM, 31.5%	MC/ lambda	Pos(in certain scattered cells)	+/+/+/ NA	Thalidomide +COP	DD (6)	(32)
10	79/M	MM	NA	NA	IgA-κ (51.4g/L)	BM ,NA	MC/kappa	Pos(B)	NA/+/+/NA	EPOCH+BB+VRD	DD (6)	(36)
Case1	54/F	non-IgM MGUS	Pos	Pos	IgG-λ (2.2g/L)	PB,NA	NA/lambda	Pos (T,B)	-/+/+/+	CHOP+ GEMOX+Selinexor+PD-1+SMILE	AWD (40)	
Case2	60/M	SMM	Neg	Neg	IgG-λ (43.8g/L)	LN,NA	MC/lambda	Pos(individual cells)	NA/+/+/NA	Lenalidomide +COP	NA	
Case3	75/M	MM	Neg	Neg	IgA-λ (11.8g/L)	BM,21%	NA/lambda	Pos(B)	-/+/+/+	Lenalidomide +CHOP	AIR (42)	

CPCP, clonal plasma cell proliferation; MGUS, monoclonal gammopathy of undetermined significance; MGRS, monoclonal gammopathy of renal significance; POEMS, Polyneuropathy, organomegaly, endocrinopathy, M-protein, skin changes syndrome; SMM, smoldering multiple myeloma; Neg, Negative; Pos, positive; NA, Not Available; BM, bone marrow; P, plasma cells; AIR, alive in remission; DD, die of disease; AWD, alive with disease; LN, lymph node; MC, monoclonal; OC, oligoclonal.

The literature search results showed numerous case reports of AITL accompanied by polyclonal plasmacytosis, and most of these cases tested negative for M protein. Two cases of AITL presenting with polyclonal plasmacytosis tested positive for clonal rearrangements of Ig in AITL samples (13, 46). We compared the retrieved case data with patient data from our own center (**Supplementary Table 1**), the PPCP group consisted of 12 cases from previous case reports (47–53). The median age of the overall patients was 69 years old, and the CPCP group showed a generally older age of the patients (median age of 70 years, range of 53–87 years old); In addition, the CPCP group had a lower LDH, with a median LDH of 310 IU/L (160–780 IU/L); The CPCP group showed significant light chain restriction (P<0.001), which is a typical hallmark of monoclonal proliferation, and leukocytosis (P = 0.005) were less frequent in the CPCP group as compared to the PPCP group; Although thrombocytopenia appeared more common in the PPCP group (75.0%) than in the CPCP group (25.0%), the difference did not reach statistical significance (p > 0.05), possibly due to the small sample size.

In terms of other clinical characteristics, the rates of gender, rash, hypoalbuminemia, bone marrow infiltration, and splenomegaly were relatively balanced between the two groups and did not show a significant differences.

The immunohistochemical expression heat map of these 25 patients is shown in (**Supplementary Figure 1**). Data analysis indicated that, when compared to the polyclonal plasma cell proliferation (PPCP) group, the clonal plasma cell proliferation (CPCP) group exhibited an older median age and a lower lactate dehydrogenase(LDH) level; however, these differences were not statistically significant (**Supplementary Table 1**). The PPCP group exhibited a higher incidence of thrombocytopenia (P>0.05), and leukocytosis (P = 0.005) compared to the CPCP group (**Supplementary Table 1**). This phenomenon appeared to be explained by the humoral immune abnormalities observed in AITL patients, which were attributed to polyclonal proliferation of plasma cells. In terms of other clinical characteristics, the rates of gender, rash, hypoalbuminemia, bone marrow infiltration, and splenomegaly were relatively balanced between the two groups and did not show differences. The analysis results revealed that light chain restriction constituted a significant differentiating characteristic between the clonal plasma cell proliferation (CPCP) group and the polyclonal plasma cell proliferation (PPCP) group (**Supplementary Table 1**). The restriction of light chains serves as a typical hallmark of monoclonal proliferation and also functions as a crucial indicator for identifying neoplastic plasma cells. Although the immunohistochemical analysis did not reveal any notable discrepancies, the findings suggested a tendency towards increased expression of CD56 and BCL6 in the CPCP group, when compared to the PPCP group. The immunohistochemical expression heat map of these 25 patients is shown in (**Supplementary Figure 1**). Early literature suggested that BCL6 was highly expressed in B cells, yet underwent a significant downregulation during the differentiation of normal B cells into plasma cells (54). In MM, BCL6 might underwent abnormal reactivation, particularly in specific subtypes such as those with low CD138 expression or IL-6-dependent cells, where heightened expression was intimately linked to tumor cell proliferation, survival, and resistance to treatment (32, 55, 56). Early studies further revealed that normal plasma cells did not express CD56, whereas malignant plasma cells demonstrate high expression of CD56, and this characteristic possessed diagnostic significance (57, 58). Our literature analysis indicated that, in addition to light chain restriction, the expression of BCL6 and CD56 also had certain significance in identifying the plasma cell characteristics within AITL tissues.

The tumor microenvironment should be an important factor in tumor immune escape and tumor occurrence. The tumor microenvironment of AITL might be an important factor leading to the malignant clonal transformation of plasma cells in AITL. Analyzing MM cases from our center and literature, we found two cases were diagnosed with MM because of monoclonal plasma cells inside the AITL. In comparison, the other three cases were diagnosed with MM because monoclonal plasma cells were detected inside the bone marrow instead of AITL. This indicated that the evolution of plasma cell tumors was not only related to the AITL microenvironment but also to the overall immune status of AITL patients.

Lenalidomide had multiple complex anti-tumor mechanisms, including anti-tumor cell proliferation, anti-angiogenesis, and immunomodulatory effects. Lenalidomide was not only the basic drug for plasma cell tumors but was also used for B-cell and T-cell lymphoma (59). Given the coexistence of AITL and plasma-cell neoplasms in our cases, lenalidomide was selected to leverage its dual therapeutic potential. Clinical data support the feasibility and activity of lenalidomide-containing regimens in AITL.

In a phase II trial of lenalidomide combined with CHOP in newly diagnosed AITL, the complete metabolic response (CMR) rate was 41% with a 2-year progression-free survival (PFS) of 42% (60). Likewise, a phase II study of CHOEP plus lenalidomide in PTCL, reported an overall response rate (ORR) of 69% and a 2-year PFS of 67% among AITL patients (61). In relapsed or refractory disease, single-agent lenalidomide demonstrated clinical activity with an ORR of about 30% (62, 63).

In contrast, bortezomib, a proteasome inhibitor, has been explored as a potential therapeutic addition to CHOP in PTCL.A multicenter phase II trial reported high initial response rates (ORR ~76%, complete remission ~65%),but long-term efficacy was limited, with a 3-year PFS of only approximately 35% (64). These findings suggest that while bortezomib-CHOP can induce rapid remissions, durability is suboptimal—likely because proteasome inhibition alone does not address the underlying IL-6/STAT3–driven survival pathways within the AITL microenvironment. In this context, lenalidomide’s established efficacy in plasma-cell disorders, together with its ability to modulate immune signaling, makes it a rational choice for composite disease. In our Case 3, lenalidomide plus CHOP achieved sustained remission, supporting this approach(Note: Case 2 data are limited by loss to follow-up). We acknowledge a theoretical risk that lenalidomide’s immune-stimulatory effects—through T-cell activation, restoration of immune-synapse function, and cytokine induction (e.g., IL-21)—could enhance Tfh-cell activity or modify the tumor microenvironment (65, 66). However, clinical data do not indicate disease acceleration in AITL, and no evidence supports that lenalidomide directly promotes Tfh-driven proliferation. Given this theoretical concern, careful monitoring of immune and viral parameters (e.g., EBV DNA load, serum M protein, and plasma-cell clonality) is recommended during treatment.

Although both lenalidomide- and bortezomib-based regimens may be considered depending on the dominant tumor component, lenalidomide-containing therapy currently appears the more balanced approach for concurrent AITL and plasma-cell neoplasms. Future studies integrating proteasome inhibitors or next-generation myeloma agents (e.g., anti-CD38 antibodies, bispecifics) may further optimize outcomes in such rare composite cases.

High-dose chemotherapy followed by hematopoietic stem-cell transplantation (HCT) remains a standard consolidation strategy for both AITL and MM in eligible patients; however, no specific guidelines exist for composite cases. In PTCL, retrospective studies support consolidative autologous HCT(ASCT) in first remission, demonstrating improved disease-free and overall survival compared with chemotherapy alone (67). Allogeneic HCT (allo-HCT)is generally reserved for relapsed or refractory cases due to its graft-versus-lymphoma potential, although it carries significant non-relapse mortality (67, 68). Similarly, in MM, ASCT remains the standard consolidation for fit patients, while allo-HCT is limited to clinical trials for high-risk or refractory disease.

For composite AITL with plasma-cell neoplasms, transplant decisions should be individualized. ASCT may be considered for eligible patients achieving remission of both components, while allo-HCT should be reserved for relapse or high-risk transformation after multidisciplinary evaluation. Early stem-cell collection is advisable, particularly in those exposed to lenalidomide, to avoid mobilization failure. Given the rarity of this dual pathology, there is a clear gap in standardized guidance. Prospective case reporting and registry studies are urgently needed to clarify optimal transplant timing and outcomes. In our cohort, none of the patients underwent HCT due to age or comorbidities, underscoring the unmet clinical need for evidence-based recommendations in this setting.

## Conclusion

In conclusion, we report 3 cases of coexistence of AITL with plasma cell tumors, including MGUS, SMM, and MM. These cases, combined with literature, demonstrated the development process of plasma cell tumors during the pathological development of AITL. Our study highlights the importance of monoclonal identification of plasma cells for accurate diagnosis and treatment planning. Moreover, immunohistochemical profiling revealed distinct expression patterns—such as elevated PD1 and CD56, and altered BCL6 expression—in clonal cases, which may serve as useful biomarkers for early recognition of plasma cell neoplasm transformation in AITL patients.

### Statistical analysis

Statistical analyses were conducted using IBM SPSS Statistics for Mac, version 29.0 (IBM Corp., Armonk, NY, USA). Continuous variables were presented as medians with interquartile ranges (IQR) due to non-normal distribution. Categorical variables were expressed as counts and percentages. Comparisons between two independent groups were performed using the Mann–Whitney U test for continuous variables, and the Chi-square test or Fisher’s exact test for categorical variables, as appropriate. All P-values were two-sided, and statistical significance was defined as P < 0.05. The statistical test used for each comparison is specified in the corresponding table and figure legends.

## Data Availability

The original contributions presented in the study are included in the article/**Supplementary Material**, further inquiries can be directed to the corresponding author/s.
