# Supplementary material for: Case Report: Angioimmunoblastic T-cell lymphoma with coexisting plasma cell tumors: three cases and review of the literature
Source: Front Oncol. 2025 Nov 21;15:1705496. doi: 10.3389/fonc.2025.1705496 (PMC12678162; doi:10.3389/fonc.2025.1705496)
Supplement: Supplementary file 1 [file Table1.docx]

| Subject | Total | PPCP | CPCP | *P* value |
| --- | --- | --- | --- | --- |
| Patient number | 25 | 12 | 13 |  |
| Age (median, range) | 69（43-87） | 68(43-87) | 70(53-87) | p˃0.05 |
| Male: Female | 15:10 | 8:4 | 7:6 | p˃0.05 |
| LDH,median(range)(IU/L)  (normal range:135-225) | 364（160-990） | 524(238-990) | 310(160-780) | p˃0.05 |
| Skin rash, +/- | 10/8 | 5/3 | 5/5 | P>0.05 |
| B symptoms, +/- | 15/4 | 9/1 | 6/3 | P>0.05 |
| hypoalbuminemia, +/- | 12/1 | 6/0 | 6/1 | P>0.05 |
| Anemia, +/- | 18/3 | 11/1 | 7/2 | P>0.05 |
| Leukocytosis,n/N(%) | 10/19(52.6%) | 9/11(81.8%) | 1/8(12.5%) | **P=0.005** |
| Thrombocytopenia,n/N(%) | 11/20(55.0%) | 9/12(75.0%) | 2/8(25.0%) | P>0.05 |
| CRP (median,range)(mg/L)  (normal range< 5 mg/L) | 13.1(0.8-46.3) | 11.1(0.8-46.3) | 15(1.3-38.7) | p˃0.05 |
| Bone marrow invasion, n/N | 5/16 | 2/9 | 3/7 | p˃0.05 |
| Splenomegaly,n/N | 14/17 | 7/7 | 7/10 | p˃0.05 |
| Light Chain Restriction,n/N(%) | 11/20(55.0%) | 0/8(0.0%) | 11/12(91.7%) | **p<0.001** |
| CD56, n/N (%) | 2/13(15.4%) | 0/9(0%) | 2/4(50%) | p˃0.05 |
| CD20, n/N (%) | 12/25(48.0%) | 5/12(41.7%) | 7/13(53.8%) | p˃0.05 |
| CD38, n/N (%) | 13/13(100%) | 8/8(100%) | 5/5(100%) | p˃0.05 |
| CD138. n/N (%) | 14/14(100%) | 8/8(100%) | 6/6(100%) | p˃0.05 |
| BCL6 n/N (%) | 9/10(90.0%) | 2/3(66.7%) | 7/7(100%) | p˃0.05 |
| PD1 n/N (%) | 7/7(100%) | 3/3(100%) | 4/4(100%) | p˃0.05 |

Supplementary Table 1. Clinical characteristics of 25 cases of AITL complicated with plasma cell proliferation.

PPCP：polyclonal plasma cell proliferation

CPCP：clonal plasma cell proliferation

+/-: Number of positive patients / Number of negative patients

n/N:Number of positive patients / Total number of patients
