# Supplementary figures and images for: Case Report: Angioimmunoblastic T-cell lymphoma with coexisting plasma cell tumors: three cases and review of the literature
Source: Front Oncol. 2025 Nov 21;15:1705496. doi: 10.3389/fonc.2025.1705496 (PMC12678162; doi:10.3389/fonc.2025.1705496)

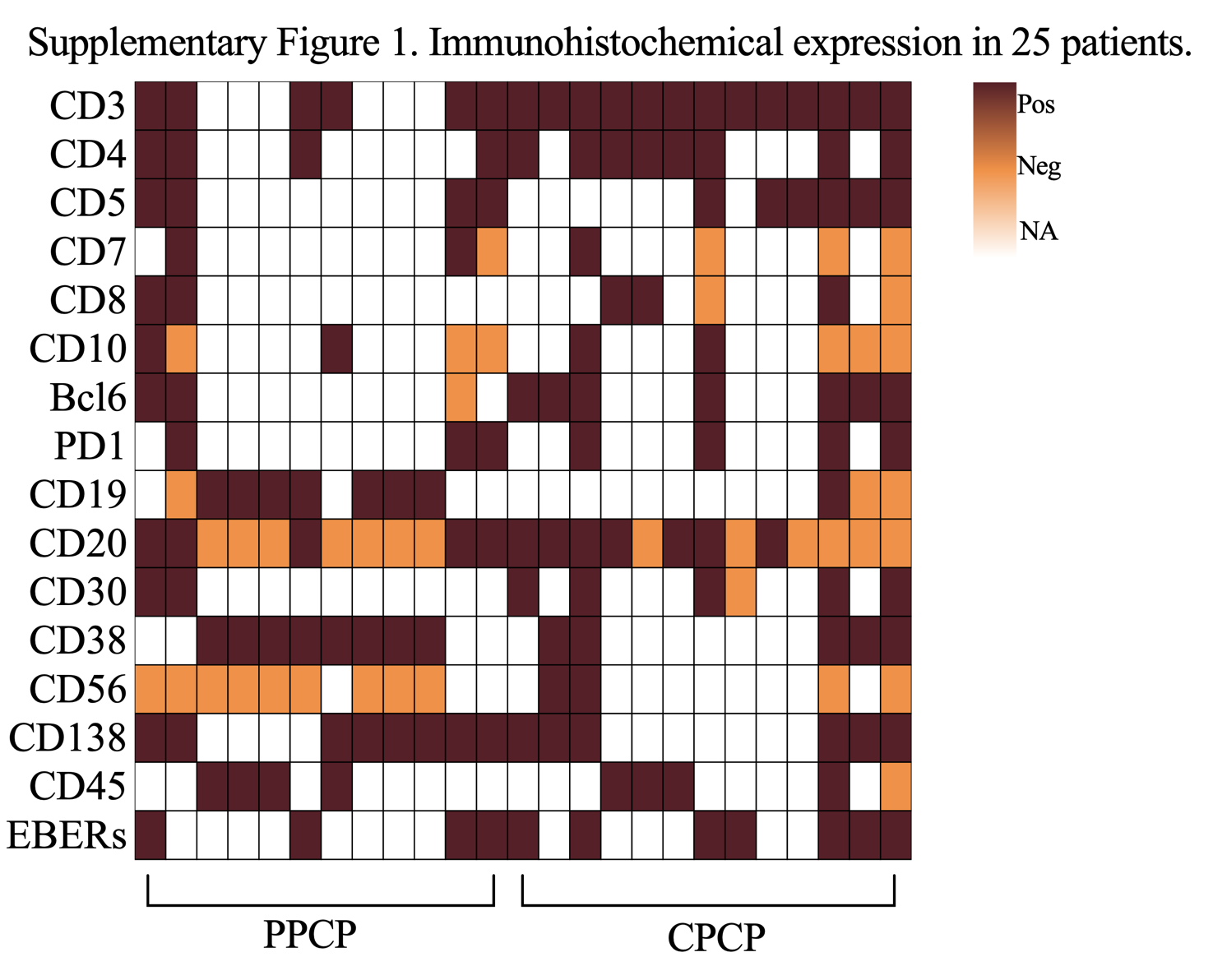

Supplement: Supplementary file 2 [file Image1.tiff]

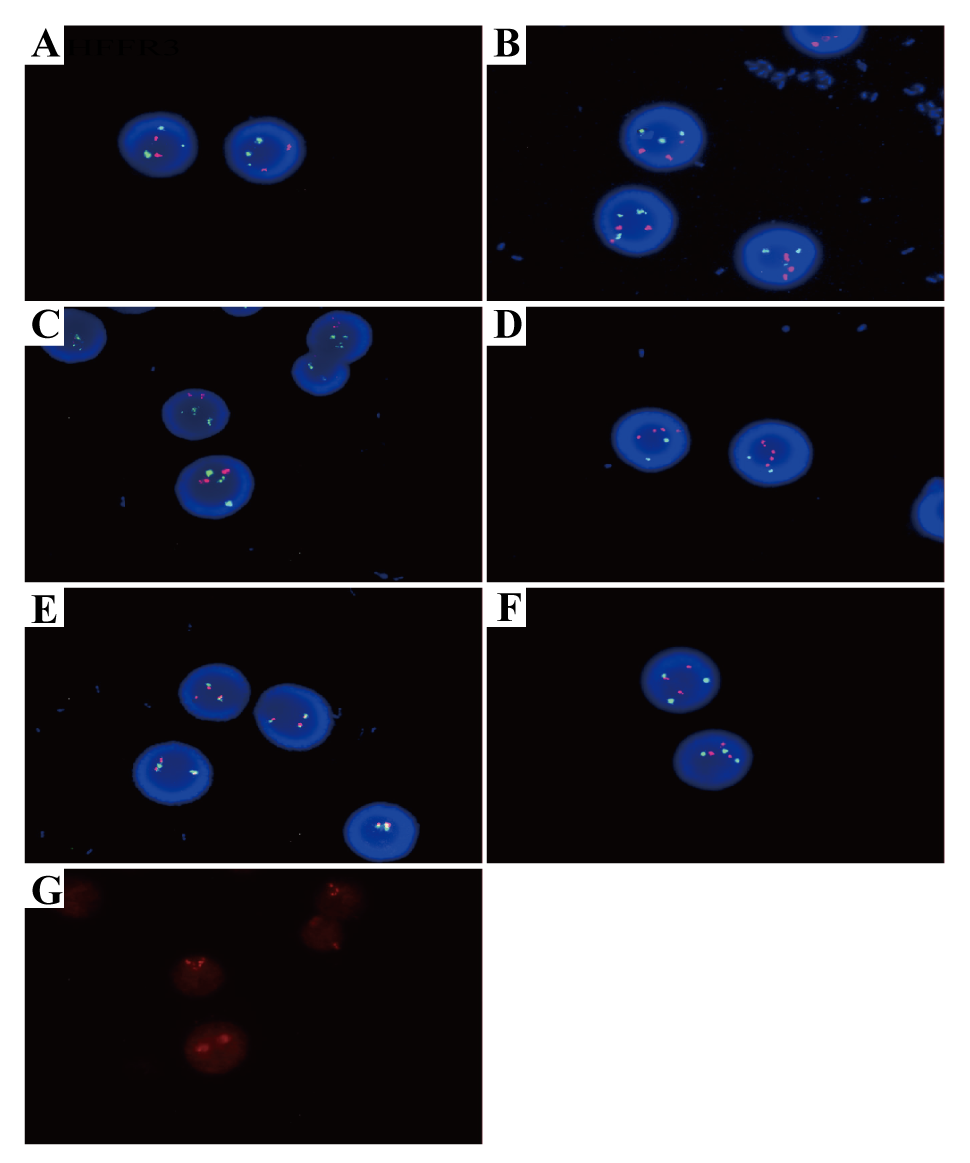

Supplement: Supplementary file 3 [file Image2.tif]

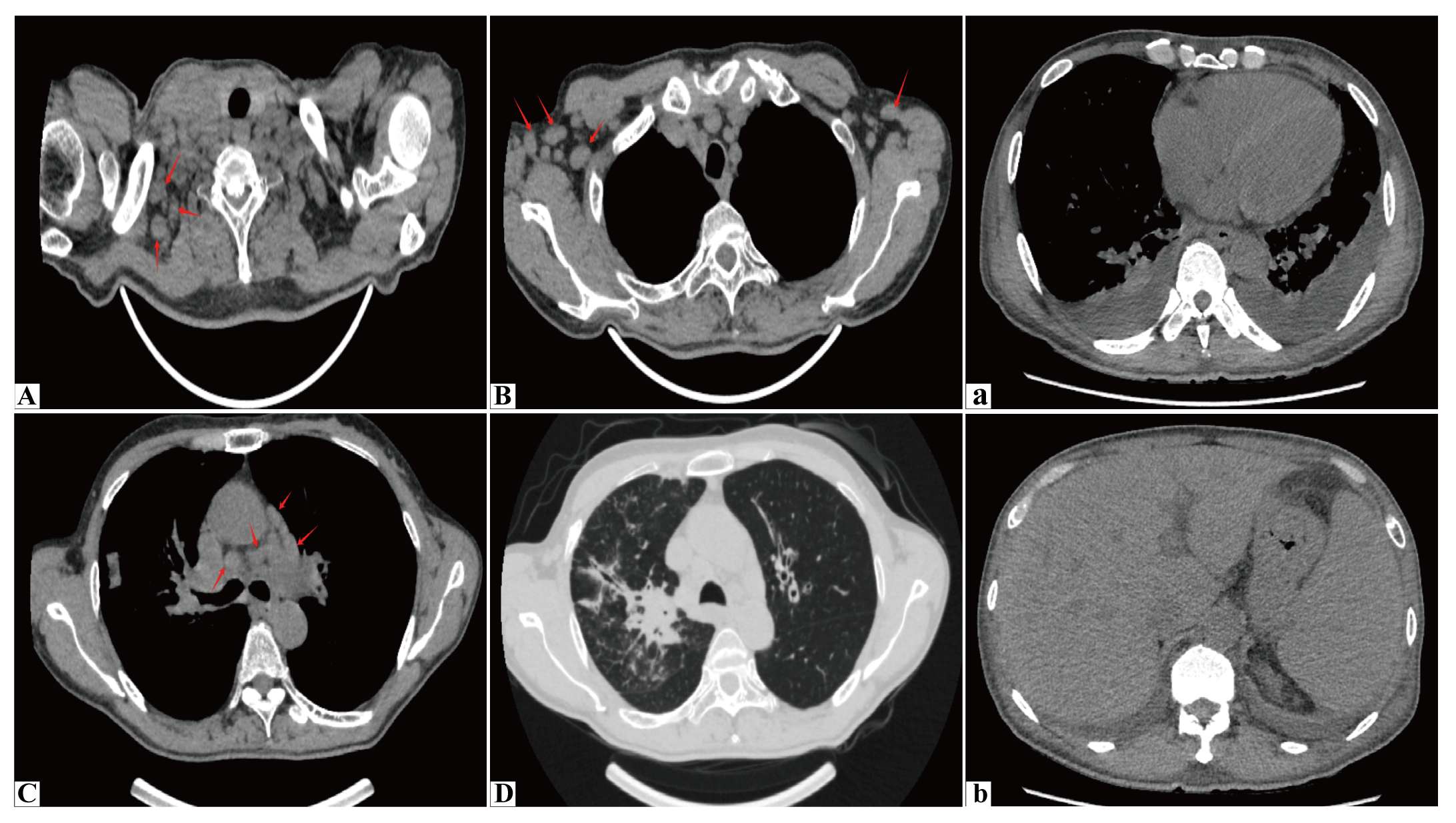

Supplement: Supplementary file 4 [file Image3.tif]

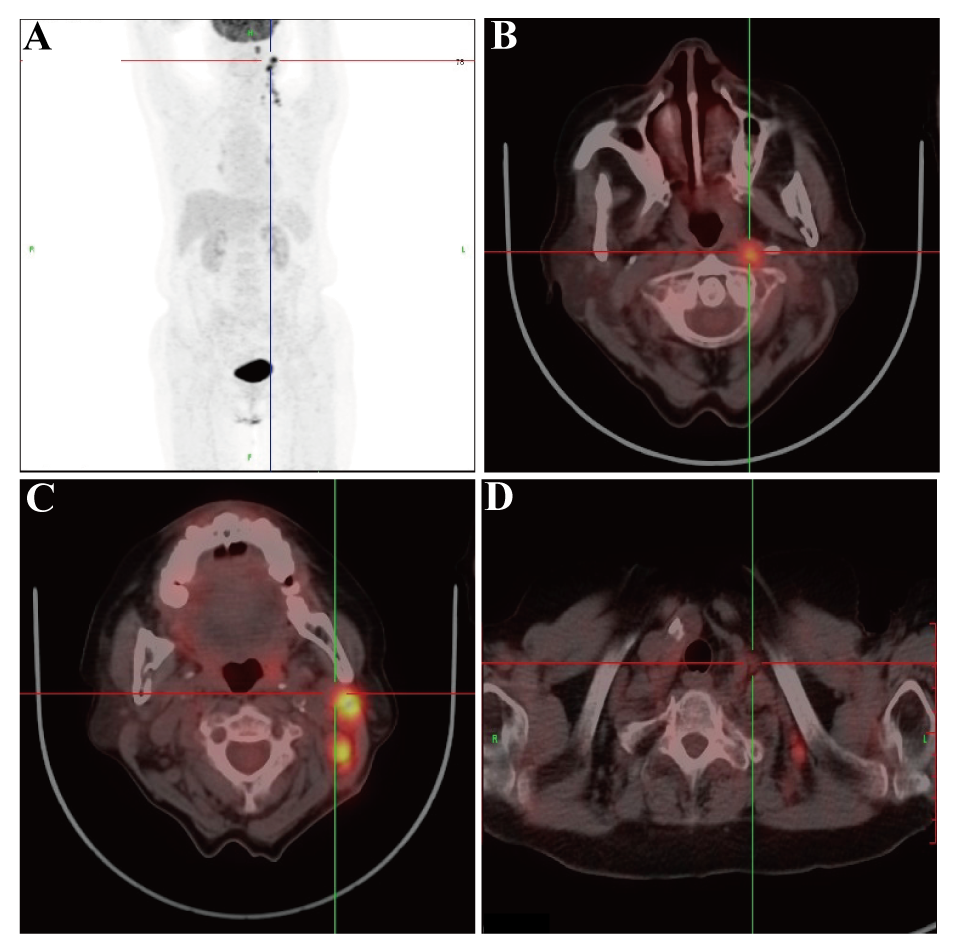

Supplement: Supplementary file 5 [file Image4.tif]
